# Strategies for large bone defect reconstruction after trauma, infections or tumour excision: a comprehensive review of the literature

**DOI:** 10.1186/s40001-021-00593-9

**Published:** 2021-10-02

**Authors:** Filippo Migliorini, Gerardo La Padula, Ernesto Torsiello, Filippo Spiezia, Francesco Oliva, Nicola Maffulli

**Affiliations:** 1 Department of Orthopaedic, Trauma, and Reconstructive Surgery, RWTH University Hospital, Pauwelsstraße 30, 52074 Aachen, Germany; 2 Department of Medicine, Surgery and Dentistry, University of Salerno, Via S. Allende, 84081 Baronissi, SA Italy; 3 Ospedale San Carlo Potenza, Via Potito Petrone, 85100 Potenza, Italy; 4 School of Pharmacy and Bioengineering, Keele University Faculty of Medicine, Thornburrow Drive, Stoke on Trent, England; 5 Barts and the London School of Medicine and Dentistry, Centre for Sports and Exercise Medicine, Mile End Hospital, Queen Mary University of London, 275 Bancroft Road, London, E1 4DG England

**Keywords:** Bone defect, Biological, Autologous, Graft

## Abstract

Large bone defects resulting from musculoskeletal tumours, infections, or trauma are often unable to heal spontaneously. The challenge for surgeons is to avoid amputation, and provide the best functional outcomes. Allograft, vascularized fibular or iliac graft, hybrid graft, extracorporeal devitalized autograft, distraction osteogenesis, induced-membrane technique, and segmental prostheses are the most common surgical strategies to manage large bone defects. Given its optimal osteogenesis, osteoinduction, osteoconduction, and histocompatibility properties, along with the lower the risk of immunological rejection, autologous graft represents the most common used strategy for reconstruction of bone defects. However, the choice of the best surgical technique is still debated, and no consensus has been reached. The present study investigated the current reconstructive strategies for large bone defect after trauma, infections, or tumour excision, discussed advantages and disadvantages of each technique, debated available techniques and materials, and evaluated complications and new perspectives.

## Introduction

Large bone defects resulting from musculoskeletal tumours, infections or trauma represent a tissue deficit unable to heal spontaneously even with adequate care and surgical stabilization [1, 2]. Surgical management aims to reconstruct the defect, avoiding amputation and providing acceptable functional outcomes [3]. Reconstruction can involve massive intercalary replacement when the whole diaphysis is replaced [4], or joint components, in association, if necessary, with prosthetic elements [5, 6]. Intercalary reconstructions showed better functional outcomes compared to other limb salvage procedures from lower morbidity of the adjacent joints [7]. Reconstructive management for large bone defects can involve autograft, allograft and non-biological materials [8]. Autografts such as vascularized fibular graft [9] or vascularized iliac bone graft [10, 11] are considered the gold standard for reconstruction of post-traumatic bone defects with non-union or malunion, given their properties of osteogenesis, osteoinduction, osteoconduction, and histocompatibility, leading to a low risk of immunological rejection [12–14]. Disadvantages are the limited amount of bone graft available, infection, prolonged wound drainage, and reoperation of the donor site [15, 16]. Another type of autograft, after bone tumour resection, consists in the re-use of the excised bone segment. For this technique, the bone segment should be processed by pasteurization, autoclaving, gamma irradiation or cryotherapy, resulting in reduction of graft osteogenicity and osteoinductivity [17–19]. Allografts represent a valid alternative to autografts, and are commonly used after bone tumour resection [20]. The main advantages of these grafts are their relative abundance, which allows reconstruction even after massive bone defect, and the absence of morbidity of the donor site [21]. The disadvantages, on the other hand, are immunological rejection and the risk of transmissive diseases, such as HIV and Hepatitis C [22–24]. However, these latter are reduced by modern screening methods [21, 23]. Bone cement spacers and induced-membrane technique is another alternative available for the reconstruction of bone defects [25, 26]. This technique is used to manage infected or uninfected bone defects [27, 28]. However, these techniques exhibit poor bone integration and slow remodelling, which make them more suitable for the reconstruction of upper limb defects, as mechanical failure is a frequent complication in the lower limb [4]. The main non-biological materials are segmental endoprosthesis [4, 21]. Segmental prostheses have several advantages, such as immediate stability, rapid rehabilitation, and early weight bearing [24]. The disadvantages, on the other hand, are infections, mechanical loosening, mechanical wear, and prosthetic and periprosthetic fracture [21, 29]. The choice of the best surgical technique is still debated, and no consensus has been reached. The present study investigated current reconstructive strategies for large bone defect after trauma, infections, or tumour excision, discussed advantages and disadvantages of each technique, debated available techniques and materials, and evaluated complications and new perspectives.

## Reconstruction using biological materials

Biological reconstructions can be managed through viable or non-viable bone material [4]. Viable bone material was considered the gold standard for reconstruction of post-traumatic bone defects from non-union or malunion, given its properties of osteogenesis, osteoinduction, osteoconduction, and histocompatibility, given its low risk of immunological rejection and a high rate of neoformation of bone [12, 13, 30]. Vascularized fibular graft, vascularized iliac bone graft, bone lengthening utilizing external fixation, and the induced-membrane technique are all examples of reconstruction techniques using viable bone [9–11]. Allograft, a biological reconstruction technique through non-viable bone, is used in reconstruction after resection of a bone tumour [6, 20, 21]. The combined reconstruction with a vascularized fibula and an allograft has the advantages of both previously described techniques [31]. Furthermore, among the reconstruction techniques which use non-viable bone material, the re-use of resected tumour bone can also be included [32]. For this purpose, the bone segment must be processed by pasteurization, gamma irradiation, or cryotherapy [17–19].

### Vascularized fibular graft

**Fig. 1:**
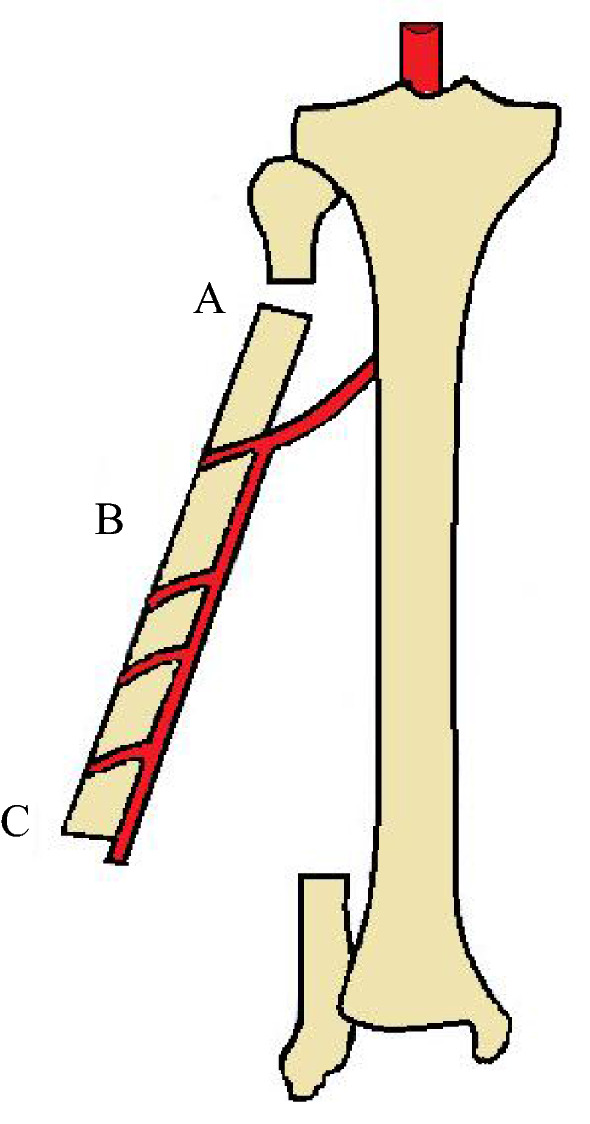
Single vascularized fibular graft with vascular pedicle. **A** Proximal osteotomy; **B** fibular graft with peroneal artery, periosteal branches, and vascular pedicle; **C** distal osteotomy

Vascularized fibular grafts are commonly used to reconstruct bone defects larger than 6 cm, after infection, tumour excision, and trauma [33], often in combination with soft tissue defects [34]. Three different options of vascularized fibular graft have been developed: single vascularized fibular graft, double-barrel technique, and combined vascularized fibula and allograft reconstruction [4, 35, 36]. In adult patients, a fibular graft up to 25–26 cm can be harvested [21, 37]. The proximal fibula and the lateral malleolus should be spared to maintain knee and ankle joint stability, protect the common peroneal nerve, and preserve weight-bearing capacity [9, 21, 33, 35, 37]. A classical single vascularized fibular graft (Fig. 1), having a reduced cross section, could be at risk of stress fractures if used in the lower limbs [34, 38, 39]. To prevent them, techniques such as the double-barrel technique and the combined vascularized fibula and allograft reconstruction have been developed [38]. Indications for single vascularized fibular graft are upper extremity reconstruction, tibial defect, bone defect in paediatric patients and in general all areas under lighter stress load [4, 40]. In the double-barrel technique, the fibular graft, thanks to its dual vascularization and adequate blood supply, can be transversely osteotomized to produce two pieces [38, 41]. This procedure, which allows the graft to double its cross section and increase its weight-bearing capacity [38, 41, 42], is particularly suitable for reconstruction of the femur and pelvis [38, 42]. However, the bone defect for which this technique can be used must not exceed 13 cm in length [41]. Complications such as infections, fixation failure and graft fracture have been reported [40, 43–45], but the rate of union was 82% at 2 years and 97% at 5 years. Union was achieved without further surgery in 70% of patients at a mean of 10 months post-operatively. Major complications such as deep soft-tissue infection, thrombosis of the pedicle, stress fracture not associated with fixation failure, compartment syndrome, and vascular injury have been reported. Liu et al. [46], in a long-term follow-up of fibular graft, reported union rates of 100% and mean union time of 21.3 weeks.

### Combined vascularized fibula and allograft reconstruction or hybrid graft

**Fig. 2:**
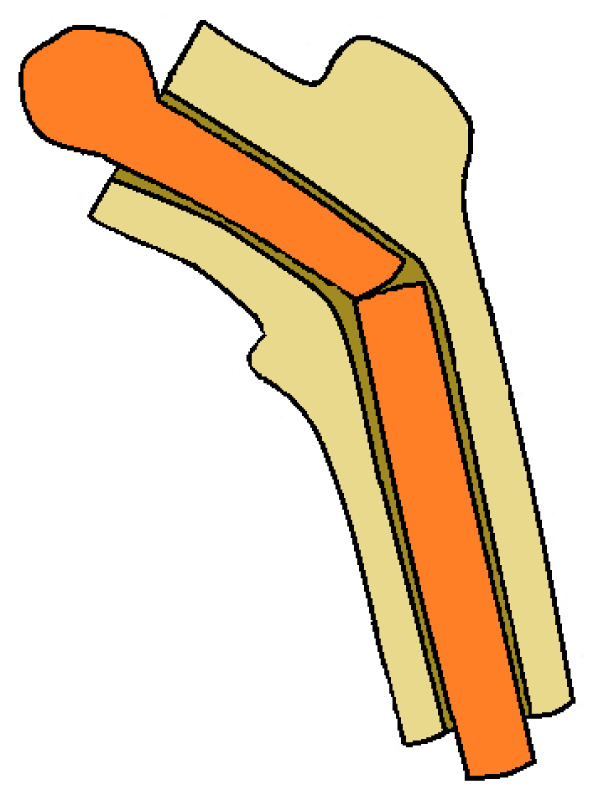
Intramedullary hybrid graft: a vascularized fibular autograft inserted into medullary canal of a cadaveric femoral allograft

First described by Capanna et al. in the early 1990s [47], this technique consists of combining the advantages provided by the mechanical strength of allografts and the advantages of the biological properties of the autograft [31, 48]. The allograft mainly provides bone support and early stability, while the vascularized fibula facilitates the integration between host and allograft, and has the ability to rapidly consolidate [31, 49, 50]. This hybrid graft can be assembled by the intramedullary or the onlay technique [50, 51]. The intramedullary technique consists of inserting the vascularized fibula inside the allograft (Fig. 2). For this purpose, the anterolateral cortex of the allograft is opened and the medullary canal reamed for a length suitable to accommodate the fibula [50–52]. When the fibula is inserted into the autograft, particular attention should be paid not to damage the vascular pedicle [52]. This technique is indicated in massive intercalary reconstruction of femur and tibia, especially in patients with long life expectancy and high physical demands [33, 50, 52]. A common complication associated with this technique is thrombosis of the anastomosed vessels, which can lead to failure of the graft [48]. Uncontrolled bleeding, stress fractures, delayed or failed bone consolidation, delayed bone growth, persistence of deformity, infections, and compartment syndrome are complications associated with the technique [9, 33, 50, 53, 54]. The overall limb salvage rate was 94%, hybrid graft fractures occurred in 23%, and infection in 6% [54]. The median time to full weight-bearing with a removable orthosis was 3.5 months, consolidation of the proximal anastomosis was achieved over a median of 6 months, four of the seven grafts fractured in the distal anastomosis between 6 and 14 months after surgery. After reoperation, consolidation of the distal anastomosis was observed after 2.8 months [50].

### ***Iliac crest bone ***graft

Iliac crest bone grafting is another commonly used strategy to reconstruct bone defects secondary to infection, tumour excision and fractures [55, 56]. Iliac crest bone graft presents all the advantages of autografts: osteogenesis, osteoinduction, osteoconduction, and histocompatibility [57–60]. The bone graft can be harvested from the anterior or the posterior iliac crest [61]. The anterior iliac crest is harvested about 4–5 cm posterior to the anterior superior iliac spine [62, 63], with a length that does not exceed 30 mm to prevent iliac crest stress fracture [64, 65]. Many techniques to harvest the anterior iliac crest have been developed, such as trapdoor technique, tricortical technique [62, 63], segmental bicortical technique, iliac crest-splitting technique, trephine technique, and acetabular reamer technique [63]. These procedures all access the inner or outer table of the ileum to harvest the cortico-cancellous graft or pure cancellous graft [63]. Accessing the posterior iliac crest allows to harvest a segment up to 30 cm long, and it is therefore indicated when a large graft volume is required. The trapdoor technique is particularly effective to obtain a cancellous graft from the posterior ileum [63]. The traditional harvesting technique to obtain an iliac graft may be associated with several complications. The complications that occur after the harvest of the autogenous iliac crest can be divided into major and minor, which occur, respectively, in 10% and 5.8% of patients [10]. Major complications are vascular injury, nerve injury, donor defect hernias, deep infection, deep hematomas, and iliac wing fractures. Minor complications are superficial infection, minor hematomas, and superficial seromas. Calori et al. [66] compared complications related to the donor site using traditional iliac crest bone graft harvest and the reamer irrigator aspirator (RIA) [60, 67, 68] technique. They noted that 14.28% of patients with iliac harvesting reported pain at the donor site, while no pain was reported by patients who underwent RIA. Infection at the donor site did not occur in any patient undergoing RIA, while 14.28% of patients undergoing iliac harvesting presented an infection. Finally, no patient operated with RIA presented a fracture at the donor site, while 2.8% of the patients who underwent iliac harvesting presented an anterior superior iliac spine fracture.

### Reconstruction by distraction osteogenesis

**Fig. 3:**
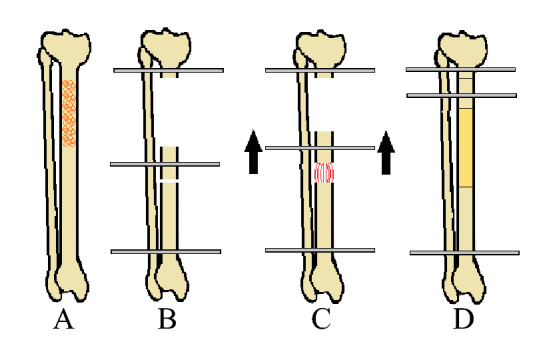
Shortening–distraction with an external fixation. **A** Osteofibrous dysplasia; **B** tumour resection, external fixation application, and osteotomy for bone transport; **C** bone transport procedure; **D** docking of bone transport and bone union

Described by Ilizarov in the 1950s [69], distraction osteogenesis has been applied to correct bone deformities, congenital musculoskeletal pathologies, manage bone defects following trauma, infections, and in cancer surgery [70–72]. In the past decades, distraction osteogenesis was seldom performed, as it was believed to cause tumour recurrence [21, 70]. Indeed, distraction osteogenesis can only be performed when full tumour resection is achieved [21, 70, 73]. Three different procedures for bone reconstruction after tumour resection have been described: segmental transport, shortening–distraction with an external fixation (Fig. 3), and shortening–distraction with an intramedullary nail to shorten the time of use of external fixation. Given the long time required for external fixation, this technique is only useful if the defect is between 4 and 6 cm [70]. Distraction begins gradually 7–14 days after surgery, with a maximum amount of 1 mm of bone distraction per day. Each millimetre of bone distraction requires approximately 2 days to consolidate [4, 21, 70]. Long-term external fixation, in addition to being uncomfortable for the patient, increases the risk of superficial and deep infections, loosening of the pins, and rotational and axial deviation [21, 70, 72, 74]. This technique regenerates living viable bone tissue to repair the bone defect [21, 75]. Schep et al. [76] reported a mean of 1.5-month distraction osteogenesis to achieve a growth of 1 cm for post-traumatic lower limb defects, and a rate of pin tract infections of 53%. Demiralp et al. [75] reported a mean time of 28 days per cm for intercalary bone defect reconstruction following bone tumour resection, and a rate of 61.5% of pin tract infections.

### Bone cement spacer and induced-membrane technique

**Fig. 4:**
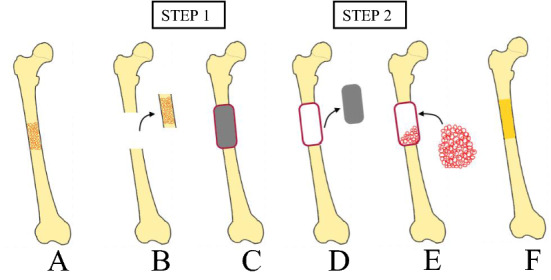
Bone cement spacer and induced-membrane technique steps. Step 1 includes debridement, fixation, cement spacer implantation and membrane formations (**A** traumatized, infected or cancerous tissues; **B** debridement; **C** spacer implantation and membrane formation); Step 2 involves in space removal preserving the membrane and the implantation of bone graft material (**D** spacer removal with membrane preservation; **E** implantation of bone graft material; **F** graft remodelling and bone regeneration)

The induced-membrane technique or Masquelet technique is used for bone defect reconstruction after infection, tumour excision and fractures [4, 25, 77]. This technique is performed in two steps (Fig. 4). The first phase consists of debridement, followed by the insertion of a polymethylmethacrylate spacer into the bone defect [78–80]. Polymethylmethacrylate causes a mild foreign-body inflammatory response which induces the development of a thick pseudo-synovial membrane which acts as a newly performed periosteum [78, 79, 81, 82]. This pseudomembrane is highly vascularized and rich in growth factors [78, 79]. The second phase, which begins after 6–8 weeks, involves opening the membrane and removing the spacer, replacing it with a bone graft [4, 25, 79, 83]. The spacer is a local antibiotic carrier, it reduces dead space, and, for this reason, it is especially indicated in the management of infected bone defects [25, 83]. However, this technique showed slow bone integration and a fair amount of graft remodelling [4, 84]. It is commonly preferred for upper limb defect reconstruction, given its high failure rate in lower limb reconstruction from mechanical failure [4]. Lemos Azi et al. [25] conducted a study on the induced-membrane technique and demonstrated that infection occurred in 68% of patients, and soft tissue reconstruction was required in 32% of patients. Bone union was achieved in 91% of patients within a mean time of 8.5 months.

### Extracorporeal devitalized autograft reconstructions

Recycling of the tumour-bearing bone segment is an alternative to allograft to repair bone defects following cancer surgery [85]. Several techniques have been developed to remove tumour cells from the resected bone fragment and make it reimplantable, including irradiation, pasteurization, and cryotherapy [18]. A most important advantage is that the processed autograft has the exact shape of the bone defect. This is particularly suitable for reconstructions in anatomically difficult tumour sites [86]. Other advantages are a low immunogenic response, absence of disease transmission, anallergicity, the no need for a bone bank, and the possibility of overcoming cultural objections to allograft found in some countries and cultures. Disadvantages include the risk of infection, fracture, bone resorption, graft necrosis, and delayed union or non-union [4, 21, 86].

### Extracorporeal irradiated autograft

Described by Spira and Lubin in 1968 [87], this technique consists of extracorporeal irradiation with reimplantation of a tumour-bearing bone [88, 89]. The first step is to remove the tumour mass with an appropriate margin, along with its surrounding soft tissues [4, 90]. Tendons and ligaments are preserved to be reused after irradiation [8, 89]. Irradiation consists of 60–70 Gy in a single dose [88, 90]. A limitation of this procedure is that the irradiated bone fragments have no blood supply and this can lead to infections, fractures, non-union, and bone re-absorption [90, 91]. To avoid this problem, a vascularized bone graft can be associated with the irradiated bone fragment, improving reconstruction and increasing vascularization [4, 89, 90]. Oike et al. [89] reported that there were no recurrences in the irradiated autograft, and 88.9% survived. Non-union occurred in 33.3%, deep infections in 14.8%, and subchondral bone collapse was observed in 14.8%. Mihara et al. [90] reported that vascularized bone grafting was successful and survived in 93.3% of patients, with 85.7% of these patients achieving complete bone union at an average of 10.8 months (range 5–24 months).

### Extracorporeal pasteurized autograft

Pasteurization consists in subjecting the tumour-bearing bone to a temperature of about 65 °C for 30–60 min, thus killing the tumour cells, without altering the osteoinductivity and mechanical resistance of the graft [92–94]. After extracorporeal pasteurization, the treated bone is relocated to its native site and fixed with plates and screws [95]. In a comparative study, among the different methods of extracorporeal devitalization of the autograft, pasteurization produced better formation of callus and better preservation of osteocytes and bone marrow cellularity [17]. The 5, 10, and 20-year survival rates of pasteurized bones, calculated using the Kaplan–Meier method, were 73%, 59%, and 40%, respectively [93]. Unfortunately, 38% of the extracorporeal pasteurized autografts were removed because of serious complications. The major complications are infection in 13% of patients, non-union in 7%, fracture of the graft in 6%, failure of fixation in 5%, resorption of the graft in 5%, and local recurrence in 4% [93].

### Extracorporeal frozen autograft

**Fig. 5:**
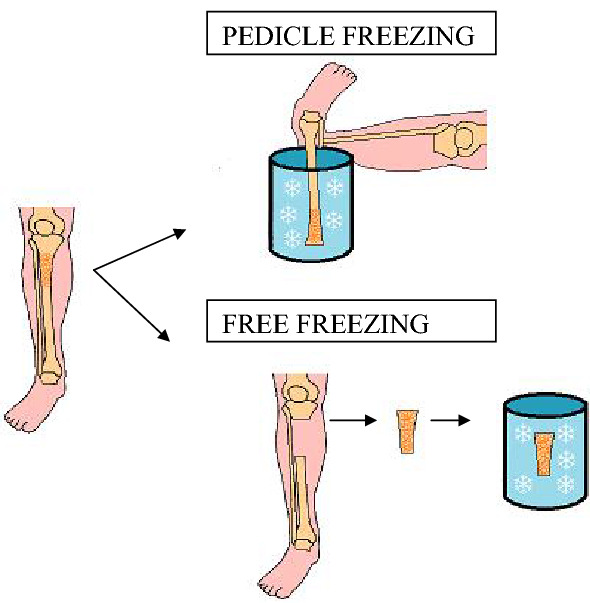
Pedicle freezing method and the free freezing method

The freezing technique uses liquid nitrogen with temperatures to –166 °C to destroy cancer cells [96]. The freezing technique induces ice crystal formation and cell dehydration [97]. Furthermore, freezing can also cause thrombosis of the microcirculation, inducing ischaemic necrosis of the tumour [4, 96, 98]. The technique involves three steps: the bone fragment is immersed for 20 min, thawed for 15 min at room temperature, and rinsed with distilled water for 10–15 min [4, 9, 99, 100]. There are two different procedures to manage the affected bone segment: free freezing and pedicle freezing technique [99] (Fig. 5). The free freezing technique involves two osteotomies [101] and soft tissue removal by immersion of neoplastic bone in liquid nitrogen [99, 100]. The pedicle freezing technique requires a single osteotomy or joint dislocation on the proximal side of the tumour-bearing bone [102]. After the osteotomy or joint dislocation, the bone is rotated and placed in a container filled with liquid nitrogen [99, 102]. Cryotherapy procedures have a number of advantages for the bone, as it retains good osteoinduction and osteoconduction properties, biomechanical strength, with no risk of disease transmission and less immunological reaction. Moreover, freezing requires less equipment than other recycling techniques [4, 96, 100]. Zekry et al. [99] reported that 5- and 10-year survival rates of frozen autografts were 91.2%, and bone union rate was 97%, but fractures occurred in 17.6% of patients, with local recurrence of disease from surrounding soft tissue in 11.8% of patients. Lu et al. [103] compared the frozen autograft with the Capanna’s technique, with no differences in the two operative procedures in terms of resection length, surgery duration, and blood loss. The mean union time for the frozen autograft was 8.4 months, significantly shorter than the Capanna’s method, which had a mean union time of 14.1 months. There are also differences between the two groups in terms of complications: infection and delayed union were observed in 6.7% and 13.3%, respectively, in the Capanna’s group, while no such complications were observed in the patients treated with frozen autograft.

## Reconstruction using non-biological materials

### Segmental prosthesis

**Fig. 6:**
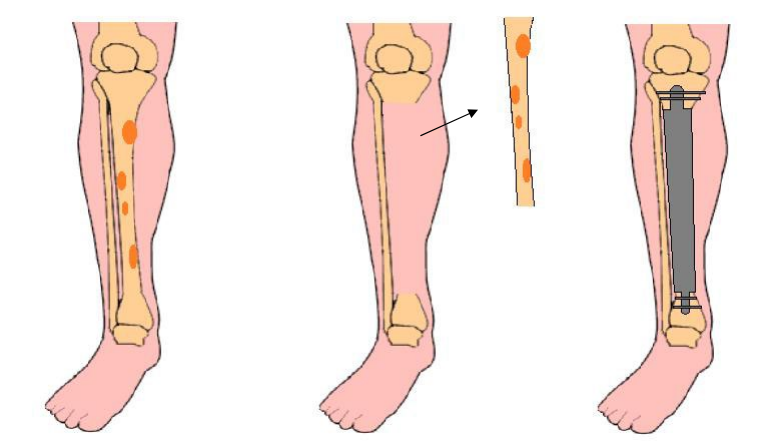
Illustration showing a large bone defect after bone tumour resection and reconstruction with a segmental prosthesis

Segmental prostheses (Fig. 6) are an alternative for intercalary reconstructions [4]. They allow immediate stability, rapid rehabilitation, and early weight bearing [24, 104]. Common complications are infections, mechanical loosening, and mechanical wear [24].

The high risk of prosthetic and periprosthetic fracture has made this technique preferred in patients with limited life expectancy from myeloma, lymphoma, or metastatic bone cancer [21, 24, 104]. Therefore, elderly patients or patients with a limited life expectancy, in whom immediate restoration of function and stability is more important than durability, are the best candidates for this technique [4, 24]. Henderson et al. [105] reported a segmental prosthesis failure rate of 24.5%, of which 49% were mechanical and 51% non-mechanical. Five failure modes were identified: the most common was infection, which occurred in 34% of patients, then failures from soft tissue problems around the implant, which were observed in 12% of patients, aseptic loosening in 19%, structural failures in 17%, and failure from tumour progression in 17%.

## Conclusions

The management of large bone defects remains a challenge. The choice of technique is still debated, and consensus is lacking. Several techniques are available to manage bone defects; however, the lack of quantitative data, along with the limited quality evidence, does not allow to infer solid conclusions. Further investigations are necessary to provide quantitative data on the rates of complication and reoperation.

## Data Availability

Not applicable.

## Abbreviation

RIA: Reamer irrigator aspirator
